# Rhizosheaths on wheat grown in acid soils: phosphorus acquisition efficiency and genetic control

**DOI:** 10.1093/jxb/erw035

**Published:** 2016-02-11

**Authors:** Richard A. James, Chandrakumara Weligama, Klara Verbyla, Peter R. Ryan, Gregory J. Rebetzke, Allan Rattey, Alan E. Richardson, Emmanuel Delhaize

**Affiliations:** ^1^CSIRO Agriculture, PO Box 1600, Canberra, ACT 2601, Australia; ^2^CSIRO Data61, PO Box 1600, Canberra, ACT 2601, Australia; ^3^Dow AgroSciences, Canberra, ACT 2601, Australia

**Keywords:** Acid soil, aluminum toxicity, genetics, heritability, phosphorus acquisition efficiency, rhizosheath, root hairs.

## Abstract

Rhizosheaths comprise soil bound to roots, and in wheat (*Triticum aestivum* L.) rhizosheath size correlates with root hair length. The aims of this study were to determine the effect that a large rhizosheath has on the phosphorus (P) acquisition by wheat and to investigate the genetic control of rhizosheath size in wheat grown on acid soil.

Near-isogenic wheat lines differing in rhizosheath size were evaluated on two acid soils. The soils were fertilized with mineral nutrients and included treatments with either low or high P. The same soils were treated with CaCO_3_ to raise the pH and detoxify Al^3+^. Genotypic differences in rhizosheath size were apparent only when soil pH was low and Al^3+^ was present. On acid soils, a large rhizosheath increased shoot biomass compared with a small rhizosheath regardless of P supply. At low P supply, increased shoot biomass could be attributed to a greater uptake of soil P, but at high P supply the increased biomass was due to some other factor. Generation means analysis indicated that rhizosheath size on acid soil was controlled by multiple, additive loci. Subsequently, a quantitative trait loci (QTL) analysis of an F_6_ population of recombinant inbred lines identified five major loci contributing to the phenotype together accounting for over 60% of the total genetic variance. One locus on chromosome 1D accounted for 34% of the genotypic variation. Genetic control of rhizosheath size appears to be relatively simple and markers based on the QTL provide valuable tools for marker assisted breeding.

## Highlight

Large rhizosheaths on wheat genotypes grown on acid soils improved the phosphorus acquisition efficiency compared with genotypes with small rhizosheaths. The rhizosheath trait was mapped to five major quantitative trait loci with largely additive genetic effect.

## Introduction

Root hairs are protrusions from single epidermal cells on a root surface that extend a plant’s influence into the surrounding soil. They are particularly important for the uptake of ions whose availability is limited by the rate of diffusion within soil. Phosphorus (P) as phosphate is poorly mobile in most soil types (Barber, 1984) and it is in the uptake of soil phosphate that the benefits of root hairs are most likely to be evident, since the hairs increase the volume of soil that can be explored. Studies that have modelled the uptake of soil phosphate by root hairs generally conclude that an increase in root hair length enhances phosphate uptake (Ma *et al.*, 2001; Leitner *et al.*, 2010; Zygalakis *et al.*, 2011).

The most compelling evidence demonstrating a benefit of root hairs to P nutrition comes from studies comparing root-hairless mutants with wild type parental lines. These studies assumed that the primary effect of the mutation was on root hairs and that pleotropic effects on other processes were minimal. In the case of the *rht3* mutant of maize (*Zea mays* L.) that lacks root hairs, grain yields in field trials were lower than wild type plants although it was not reported whether this was a consequence of altered uptake of water and nutrients or if it could be attributed to a pleotropic effect (Hochholdinger *et al.*, 2008). Nevertheless, several studies have shown that mutants of barley (*Hordeum vulgare* L.) lacking root hairs have reduced phosphate uptake compared with wild type plants in low P treatments in soil culture and this is associated with decreased biomass production (Gahoonia *et al.*, 2001; Gahoonia and Nielsen, 2003; Chen *et al.*, 2005; Zheng *et al.*, 2011; Brown *et al.*, 2012; Haling *et al.*, 2013). Similarly, *Arabidopsis thaliana* mutants that lack root hairs were smaller than wild type plants when grown under restricted P supply in a sand–alumina mix but were the same size as wild type with a high P supply (Bates and Lynch, 2000, 2001).

Although these studies clearly demonstrate the benefit of root hairs for phosphate uptake, it is not clear whether variation in root hair length within a species can be exploited to improve phosphorus acquisition efficiency (PAE). In contrast to mutants, the germplasm will all possess root hairs and differ only in their length or density whereas the mutants represent an extreme comparison. By comparing genotypes that differ in root hair length and density within a species, several studies have shown that root hair length is correlated with P uptake and biomass accumulation under low P supply (Gahoonia *et al.*, 1997; Krasilnikoff *et al.*, 2003; Zhu *et al.*, 2010; Vandamme *et al.*, 2013) and in at least one instance, with final grain yield (Gahoonia and Nielsen, 2004). However, in all these examples unrelated genotypes were compared and the genotypes may have differed in additional root traits that contributed towards differences in PAE.

A previous study described wheat lines that differ in root hair length when grown on acid soil and this was attributed to differences in the ability of root hairs to tolerate Al^3+^ in the soil solution (Delhaize *et al.*, 2012a). In that work the rhizosheath (soil adhering to the root) size of young seedlings was strongly correlated with length of root hairs and was used as a surrogate for root hair length to develop backcrossed germplasm. The germplasm comprised near-isogenic lines that differed in rhizosheath size, and hence length of root hairs, when grown on an acid soil. These differences in rhizosheath size either disappeared or were reduced when the same soil was limed to a higher pH (Delhaize *et al.*, 2012a). To date, the genetics of rhizosheath size of wheat grown on acid soil is unexplored even though near-isogenic lines differing in rhizosheath size have been developed (Delhaize *et al.*, 2012a). Similar to wheat grown on acid soil, root hair length of wheat grown on non-acid soil was strongly correlated with rhizosheath size (Delhaize *et al.*, 2015). Mapping quantitative trait loci (QTL) for rhizosheath size on non-acid soil identified six major loci together accounting for 42% of the variation in rhizosheath size (Delhaize *et al.*, 2015). George *et al.* (2014) screened a diverse population of barley genotypes on non-acid soil and by genome wide association analysis identified loci on chromosomes 2H, 5H and 7H as contributing to rhizosheath size. Unlike wheat, rhizosheath size of barley was not strongly correlated with root hair length.

Acid soils limit crop production on large tracts of agricultural land globally, primarily due to Al^3+^ toxicity but also due to P deficiencies caused by the formation of Al–P complexes (von Uexküll and Mutert, 1995). The use of wheat germplasm with Al^3+^-tolerant roots improves PAE on acid soils, but root hair elongation can still be inhibited on these roots (Delhaize *et al.*, 2009). As discussed above, there is strong evidence that root hairs are important for PAE on non-acid soils. The use of germplasm that differs in root hair length on acid soil will help establish whether root hair length is also important for PAE on acid soil. Here we use near-isogenic lines (NILs) that differ in Al^3+^ tolerance of root hairs to show that large rhizosheath size on acid soils is associated with improved PAE. In addition, we undertook genetic analyses to establish heritability and to determine the number and chromosomal location of loci controlling rhizosheath size of wheat grown on acid soil. Markers linked to the QTL identified from the analysis can be applied to breed wheat for improved PAE on acid soils.

## Materials and methods

### Germplasm

NILs of wheat differing in Al^3+^ tolerance of root hairs using rhizosheath size on acid soil as a surrogate for root hair length were generated previously (Delhaize *et al.*, 2012a). Briefly, the Brazilian cultivar Fronteira with a large rhizosheath was crossed to the Australian cultivar EGA Burke as the small rhizosheath recurrent parent (Delhaize *et al.*, 2012a). Both EGA Burke and Fronteira possess an Al^3+^-tolerant allele of *TaALMT1*, a gene that confers Al^3+^ tolerance to root growth. Three backcrosses into EGA Burke were completed after phenotypic selection of the F_1_ at each generation. BC_3_F_4_ lines with consistently large (L1, L2, L4, L5) or small (S2, S3, S4) rhizosheaths were identified after selections at the BC_3_F_2_ and BC_3_F_3_ generations and evaluation at the BC_3_F_4_ generation using the rhizosheath phenotypic screen. We refer to the BC_3_ lines with Al^3+^-tolerant root hairs identified from screens on acid soil as large (L) rhizosheath lines whereas those with Al^3+^-sensitive root hairs are referred to as small (S) rhizosheath lines.

For the generation means analysis (GMA) Fronteira was crossed to either EGA Burke or Yitpi as outlined below. Yitpi possesses the same Al^3+^-tolerant allele of *TaALMT1* as Fronteira and EGA Burke and this *TaALMT1* allele can be considered to be genetically ‘fixed’ in all germplasm used in the experiments. *TaALMT1* is the major Al^3+^ tolerance gene of wheat and encodes an anion channel facilitating the efflux of malate from roots (Delhaize *et al.*, 2012b). For QTL analysis, a population of 139 F_6_-derived, F_7_ recombinant inbred lines (RILs) was developed by single-seed descent from an EGA Burke by Fronteira cross.

### Rhizosheath screens

Rhizosheath screens were conducted in controlled environment growth cabinets according to a previously described method (Delhaize *et al.*, 2012a) with soils used in the PAE experiments (Table 1). Briefly, the air-dried soil with mineral nutrients added as described below was sieved through a 4mm mesh, and water was added to 80% of field capacity (FC), which was 28.8% for the ferrosol and 16.0% for the kandosol. The moistened soil was mixed manually and again sieved through a 4mm mesh. The soil was packed to 250±3g into small pots of 5.4cm width and 9.5cm height to a bulk density of about 0.80g cm^−3^. After sowing a single pre-germinated seed, pots were placed in trays covered with transparent plastic lids. Air temperature in the growth cabinet was maintained at 23 °C, humidity maintained at about 70%, and light intensity set at 100 µmol m^−2^ s^−1^ photon irradiance with an 8h photoperiod. Intact seedlings were harvested after 3 d when leaf 1 was about two-thirds extended to its final length. The three primary seminal roots were excised from seedlings and weighed together with adhering soil. Root length was then measured and rhizosheath calculated as the fresh weight of soil and root per length of seminal root.

**Table 1.** Characterization of P-responsive soils used in rhizosheath screening experiments and glasshouse growth trials

Parameter	Ferrosol^*a*^(Robertson)	Kandosol^*a*^(Rye Park)

pH	4.3	4.0
Field capacity (%w/w)	36.0	20.0
Colwell P (mg P kg^−1^)	41.7	8.0
PBI^*b*^	1117	70
Total P (µg g^−1^)	1260	89
Soluble Al	27.6	40.0

a Soil classified according to Isbell (1996).

b Phosphorus buffer index.

### Short term growth experiments on P-limiting soils

#### Soil characterization and treatments

A ferrosol and a yellow kandosol (Isbell, 1996) were collected from farmers’ paddocks in southern New South Wales at Robertson (34°35′S, 150°36′E) and Rye Park (34°31′S, 148°55′ E), respectively, from below the 10cm soil layer. Air-dried soils were passed through a 4-mm sieve. Treatments consisted of two rates of P where the ferrosol received 250 and 2000mg kg^−1^ and the kandosol 50 and 150mg kg^−1^. The lower P rate for each soil type was considered to be a rate that was not severely P-deficient for growth of wheat yet was responsive to P application for growth, whereas the higher P rate was considered to be non-limiting for plant growth. The ferrosol is a highly P-fixing soil as seen by its much greater phosphorus buffer index (PBI) than the kandosol (Table 1), and required larger amounts of applied P than the kandosol for adequate shoot growth at both low and high treatments. Phosphorous was applied to the air-dry soil as finely ground KH_2_PO_4_ (22.8% P w/v) and mixed well with the soil prior to the addition of the nutrient solution. The soil was brought up to 85% of moisture field capacity by mixing with nutrient solution (6.5mM KNO_3_, 2mM Ca(NO_3_)_2_, 3mM (NH_4_)_2_SO_4_, 2mM MgSO_4_, 45 µM FeCl_3_, 23 μM H_3_BO_3_, 5 μM MnCl_2_.4H_2_O, 2 μM ZnSO_4_.7H_2_O, 1 μM (NH_4_)_6_Mo_7_O_24_.4H_2_O and 2 μM CuSO_4_.5H_2_O) in a low-geared cement-mixer just prior to packing of pots. When required, lime was applied to the soils at a rate of 4g kg^−1^ to increase the pH from 4.3 to about 5.5 in the ferrosol and from 4.0 to about 5.5 in the kandosol.

Soil was packed into cylindrical pots (10.5cm internal diameter and 20cm height) to a bulk density of 0.90g cm^−3^ for the ferrosol and 1.3g cm^−3^ for the kandosol. The soil bulk densities were chosen to be similar to the bulk densities used in the screening of germplasm for rhizosheath size. Each pot contained between 1200 and 1700g dry soil (depending on bulk density), which was packed to a depth of 17cm. Soil strength measured using a penetrometer with a cylindrical rod diameter of 0.625cm (0.307cm^2^) ranged from 0.2 to 0.3MPa.

Field capacity of soils was determined to be 36.0% and 20.0% moisture content (gravimetric) for the ferrosol and kandosol, respectively, using the wetting-front method described by Passioura (2006). Gravimetric moisture contents of soils were determined after drying for 48h at 105 °C. Soil characteristics are summarized in Table 1. Soil P was extracted in 0.5M NaHCO_3_ adjusted to pH 8.5 with 5M KOH (soil solution ratio of 1:100 and extraction time of 16h at 25 °C) according to the Colwell method (1963). The total P in soils was determined on soil samples heated in a muffle furnace at 550 °C for 4h, and after cooling the soil was subsequently extracted in 0.5M H_2_SO_4_ (Saunders and Williams, 1955). Phosphate in extracts for Colwell and total P were determined by the malachite green method (Irving and McLaughlin, 1990). PBI was measured using previously described methods (Burkitt *et al.*, 2008; Rayment and Lyons, 2010). Soil pH was measured in 0.01M CaCl_2_ soil extracts (1:5 w/v soil:solution ratio) where samples were shaken for 1h prior to centrifugation and collection of the supernatant solution.

#### Plant growth

To determine the effect that a large rhizosheath had on P acquisition and subsequent shoot growth in P-limiting soils, shoot growth of the NILs differing in rhizosheath size were evaluated in two contrasting low pH, P-limiting soils containing toxic concentrations of soluble Al^3+^ (Table 1). In separate experiments, growth of the germplasm was also evaluated on the same soils amended with lime to raise the pH from 4.3 to 5.5. In all experiments, two P treatments were incorporated into the soils: a high P rate estimated to be non-limiting to shoot growth and a responsive P rate that limited growth without causing severe P deficiency.

Grains of individual lines were selected within a 5mg weight range (55–60mg), imbibed overnight at 4 °C and then germinated on filter paper in Petri dishes over 2 d. Germinated grains were planted one per pot to a depth of about 1cm, and the soil surface covered with a 2cm layer of white plastic beads to reduce evaporation. The plants were grown under naturally lit glasshouse conditions at CSIRO, Canberra, Australia (35°16′S, 149°7′E) at air temperature maintained at approximately 25 °C (day) and 15 °C (night). Experiments were conducted over July to September 2012 for the ferrosol (both acid and limed), over October to November 2012 for the acid kandosol and over March to April 2013 for the limed kandosol. Pots were watered to weight with deionised water to 85% of field capacity every 2 d. The experiments were arranged in a factorial design with two P treatments and six or seven wheat genotypes, and were run in four replicate blocks. Data were analysed using ANOVA (SigmaPlot version 12.3) to generate means and least significant differences (LSDs).

#### Shoot harvest and P determination

Shoots were harvested at 26 (acid soil) or 24 (limed soil) days after emergence for experiments with the ferrosol and 24 (acid soil) or 21 (limed soil) days after emergence for growth experiments using the kandosol. Shoots were dried at 70 °C for 48h and weighed. Dried shoots were milled to a fine powder using a puck mill for determination of total P. Briefly the samples (about 50mg) were ignited in a muffle furnace at 550 °C for 5h. The ashed samples were subsequently dissolved in 5ml of 2M HCl and phosphate concentration determined by a modified malachite green method (Murphy and Riley, 1962). Shoot P content was calculated as the product of shoot dry weight and P concentration in the shoot.

### Genetics of the rhizosheath trait

#### Generation means analysis

Experiments were conducted to investigate the genetic control of the rhizosheath trait in several wheat populations. In view of the low heritability common for root traits, gene action was first investigated with the generation means mating design based on first-order statistics. The cultivar Fronteira with a large rhizosheath was crossed to the smaller rhizosheath cultivars EGA Burke and Yitpi to produce an F_1_ generation for each population. Fronteira was used as the female parent in all crossing although reciprocal crosses using Fronteira as the male parent were also undertaken to assess maternal genetic effects in reciprocal F_1_ grains. Four F_1_ grains were sown and plants self-pollinated to produce F_2_ generations, whereas other F_1_ plants were backcrossed to each of the original parents to develop BC_1_F_1_ generations. For each population, approximately 20 grains were sown of each parent and F_1_, and 10 of each F_1_ reciprocal cross. For the various generations, 180 F_2_, 42 BC_1_P_1_ and 42 BC_1_P_2_ grains were sown for each population where P_1_ and P_2_ represent each of the parents.

To estimate gene effects for rhizosheath size in each population, weighted least squares regression analyses were used to solve for the mid-parent (*m*), pooled additive ([*a*]), pooled dominance ([*d*]) and pooled digenic epistatic ([*aa*], [*ad*], and [*dd*]) genetic effects following the models and assumptions described in Mather and Jinks (1971). A simple additive-dominance genetic model containing only *m*, *a*, and *d* effects was first tested using the joint scaling test (Rowe and Alexander, 1980). Adequacy of the genetic model was assessed using a chi-square goodness-of-fit statistic determined from deviations from the additive-dominance genetic model for each experiment and then pooled across experiments. Broad- and narrow-sense heritabilities (and their standard errors) were calculated (Ketata *et al.*, 1976) and numbers of effective factors were then estimated (Falconer and Mackay, 1996). In addition, a separate, random sample of 200 F_2_ seed from the Fronteira/EGA Burke population was grown along with parents and assessed for rhizosheath size. Tails of this population comprising the seedlings with the smallest and largest 10% of rhizosheaths within the F_2_ population were selected, transferred to pots and grown to maturity. The resulting F_3_ generation of these selections was assessed for rhizosheath size, and means calculated and realised heritability estimated according to Falconer and Mackay (1996). All statistical analyses were undertaken using the SAS mixed-linear modelling procedure Proc MIXED (SAS, 2013).

#### QTL analysis

Rhizosheath size data collected on the EGA Burke × Fronteira RIL population were analysed using AsReml-R (Butler *et al.*, 2011) after first checking for normality. The analysis took into consideration the experimental design which used a nested blocking structure in which the factor Tray (eight levels) was nested in Replicate (six levels). QTL analysis was carried out using whole genome average interval mapping (WGAIM) as described by Verbyla *et al.* (2007) and extended in Verbyla *et al.* (2012). The genetic map used in the QTL analyses was obtained from the RIL and parental lines by analysis with the 90K SNP chip (Wang *et al.*, 2014). These markers were mapped to 35 linkage groups yielding a total map length of 9375.72 cM. Since many of these markers were located at the same position on the map, a set of markers was removed to ensure non-zero recombination fractions between the remaining markers. The final map for QTL analysis consisted of 2332 markers.

## Results

### Characterization of germplasm

**Fig. 1.**
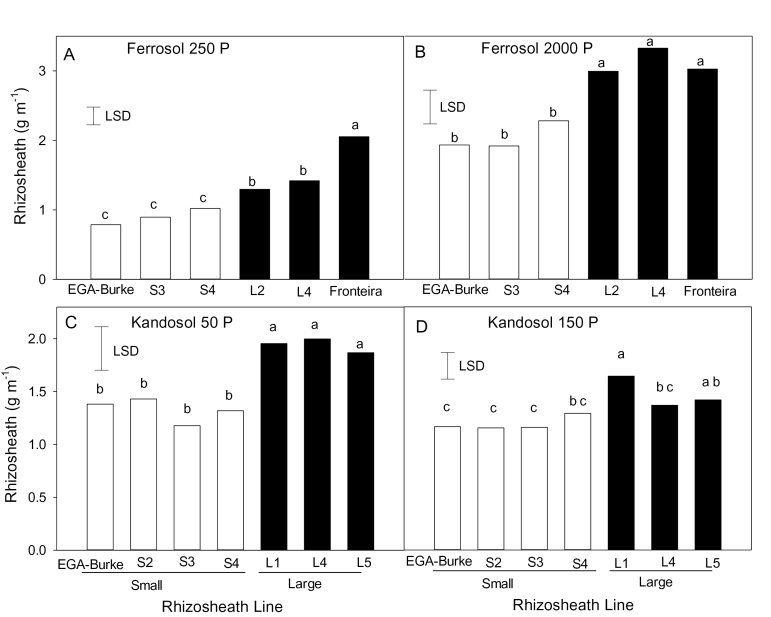
Rhizosheath size on EGA Burke, Fronteira and selected BC_3_ NILS with small (S lines, empty bars) or large (L lines, filled bars) rhizosheaths on an acid ferrosol (A, B) and an acid kandosol (C, D) with a responsive (A, C) or a non-responsive (B, D) P rate added to the soils. Values are means (*n*=6) and different letters indicate significant differences (*P*<0.05) between genotypes.

To verify the rhizosheath traits, the L and S lines together with parents EGA Burke and Fronteira were screened on two acid soils that had been fertilized. One of these soils was a low pH ferrosol containing toxic concentrations of Al^3+^ (Table 1), a soil that had previously been used to develop the L and S rhizosheath lines (Delhaize *et al.*, 2012a). In the lower P responsive treatment (250mg kg^−1^), rhizosheath size of S lines was similar to the recurrent parent EGA Burke (Fig. 1A). Conversely, the rhizosheath size of L lines was significantly larger than both EGA Burke and S lines, but smaller than that of donor parent Fronteira. While the rhizosheath size of all lines increased by a factor of about 2 with a higher rate of applied P (2000mg kg^−1^), the ranking of lines remained similar except that the size of the rhizosheath of the L lines was now comparable to that of Fronteira (Fig. 1B). Similarly, in the acid kandosol, rhizosheath sizes of L lines were about 40% greater on the low P soil than those of S lines (Fig. 1C). Differences in rhizosheath size between lines were still apparent, although attenuated, at the higher P rate (150mg kg^−1^) in the kandosol (Fig. 1D). When the soil pH values of the ferrosol and kandosol were adjusted to 5.5 by the application of lime, rhizosheath size of all lines increased for both P treatments and differences in rhizosheath size between the different lines essentially disappeared (see Supplementary Fig. S1 at *JXB* online).

### Short term growth experiments on P-limiting soils

**Fig. 2.**
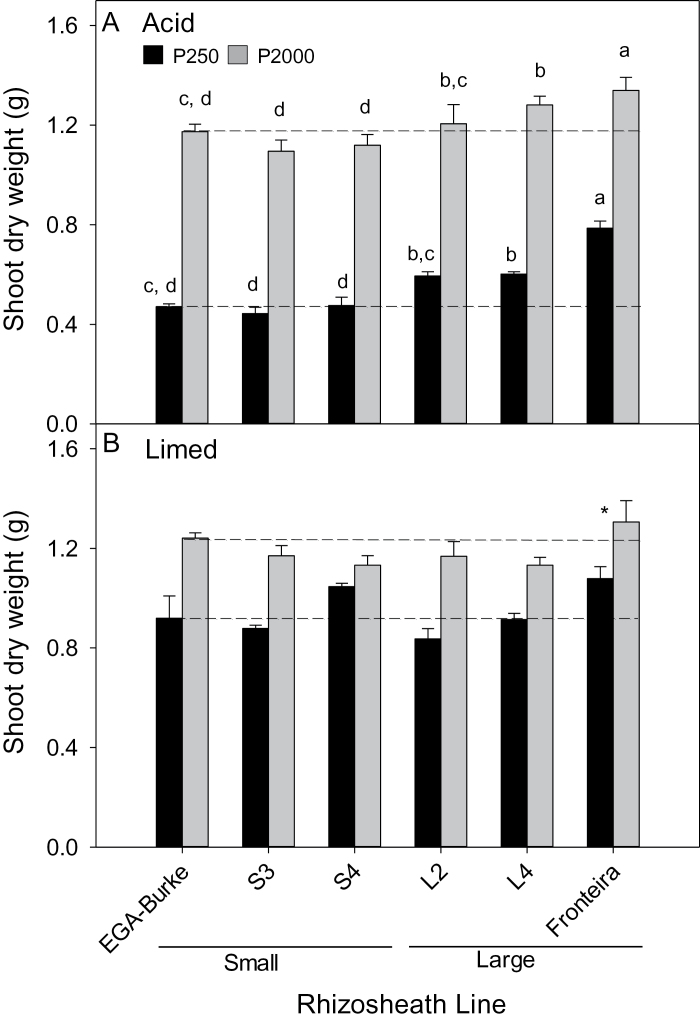
Shoot biomass of EGA Burke, Fronteira and BC_3_ NILS with large (L2, L4) or small (S3, S4) rhizosheaths grown in (A) a non-limed (pH 4.3) and (B) a limed (pH 5.5) ferrosol with 250mg kg^–1^ or 2000mg kg^–1^ added P. Values are means (*n*=4) and different letters indicate significant differences (*P*<0.05) between genotypes (no interaction between genotypes and treatments). A significant genotype difference (*P*<0.05) in (B) is indicated by *. For reference, the dashed lines show shoot dry weights of EGA Burke at low (lower lines) and high (upper lines) P treatments.

**Fig. 3.**
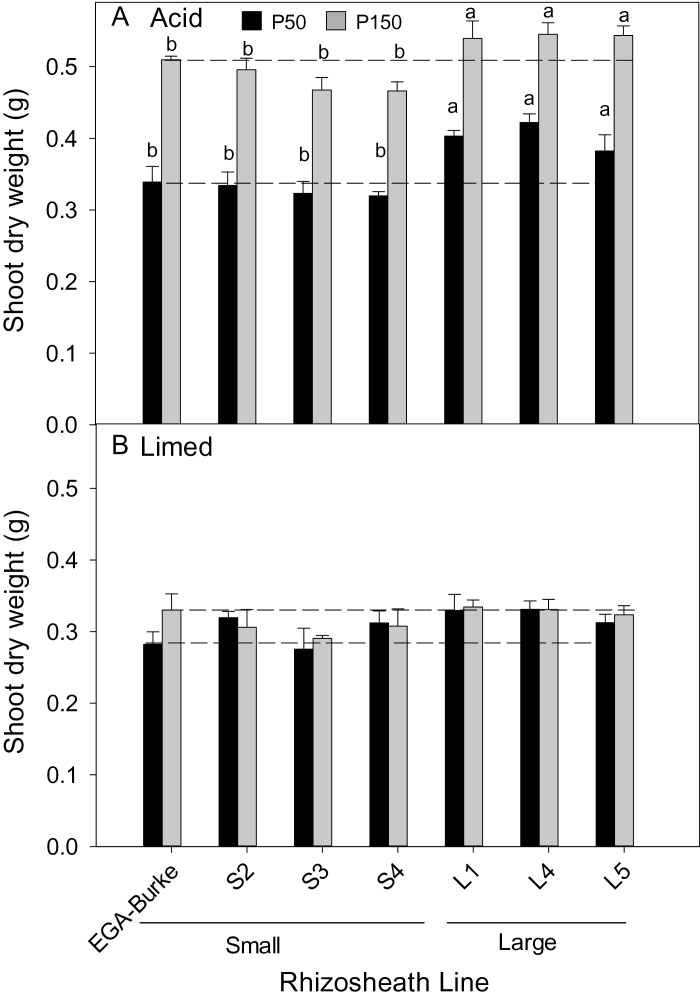
Shoot biomass of EGA Burke and BC_3_ NILS with large (L1, L4, L5) or small (S2, S3, S4) rhizosheaths grown in (A) non-limed (pH 4.0) or (B) limed (pH 5.5) kandosol with 50mg kg^−1^ or 150mg kg^−1^ added P. Values are means (*n*=4) and different letters indicate significant differences (*P*<0.05) between genotypes (no interaction between genotypes and treatments). For reference, the dashed lines show shoot dry weights of EGA Burke at low (lower lines) and high (upper lines) P treatments.

**Fig. 4.**
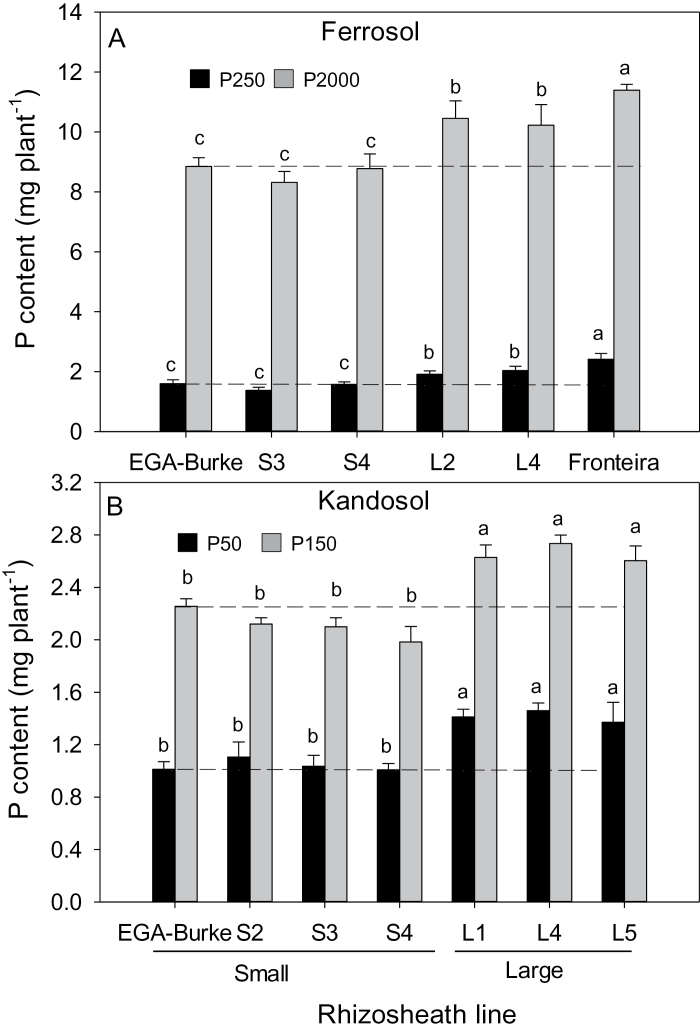
Shoot P content (total P accumulated) in shoots of EGA Burke and BC_3_ NILS with large (L) or small rhizosheaths (S) grown in (A) acid ferrosol (pH 4.3) with 125mg kg^−1^ or 2000mg kg^−1^ added P or (B) acid kandosol (pH 4.0) with 50mg kg^−1^ or 150mg kg^−1^ added P. Values are means (*n*=4) and different letters indicate significant differences (*P*<0.05) between genotype means (no interaction between genotypes and treatments). For reference, the dashed lines show shoot P content of EGA Burke at low (lower lines) and high (upper lines) P treatments.

**Fig. 5.**
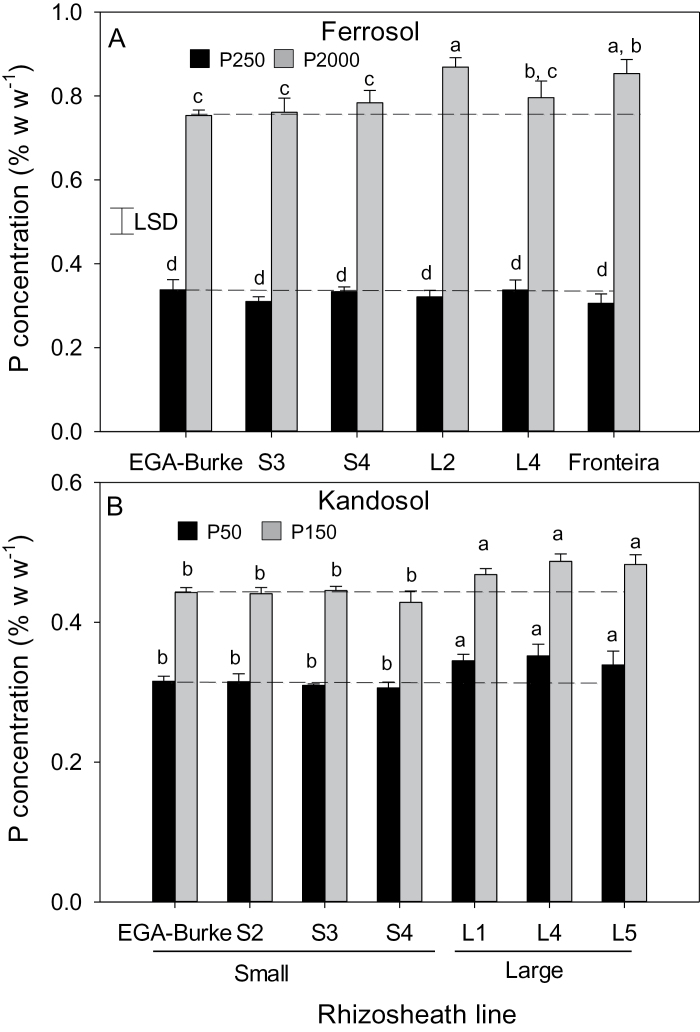
Shoot P concentrations of EGA Burke and BC_3_ NILS with large rhizosheath (L) or small rhizosheath (S) grown on (A) an acid ferrosol (pH 4.3) with 250mg kg^–1^ or 2000mg kg^–1^ added P or (B) an acid kandosol (pH 4.0) with 50mg kg^–1^ or 150mg kg^–1^ added P. Values are means (*n*=4). For (A) there was a significant interaction between P treatment and genotype and the LSD is shown along with different letters to indicate significant differences (*P*<0.05) between genotype means and treatments. For (B) there was no interaction between genotype and P treatment and the different letters denote genotypes that differed significantly with *P*<0.05. For reference, the dashed lines show shoot P concentrations of EGA Burke at low (lower lines) and high (upper lines) P treatments.

For plants grown on both acid soils, the L lines generally had greater shoot biomass than S and parental EGA Burke lines regardless of P treatment (Figs 2A and 3A). When the soils were limed to increase the pH from 4.3 to 5.5, the differences between S and L rhizosheath line soils disappeared (Figs 2B and 3B). The differences in shoot biomass on the acid soils were reflected in the amounts of P accumulated in shoots, with EGA Burke L lines accumulating more P than S lines on both soils regardless of P treatment (Fig. 4). When the ferrosol was limed, shoot P concentrations and shoot P content of the lines did not differ from one another at the low P treatment and any differences apparent between the lines at the high P treatment were not consistently associated with rhizosheath size found on acid soil (see Supplementary Table S1 at *JXB* online). The P concentrations in shoots of L and S lines grown in the low P treatment of the acid ferrosol did not differ from one another (Fig. 5). In the high P acid ferrosol, one of the L lines as well as Fronteira had significantly (*P*<0.05) greater P concentrations than all the S lines and EGA Burke. For the acid kandasol, all L lines had greater P concentrations than S lines regardless of the P supply (Fig. 5). The soil P was unlikely to have been limiting growth in the high P treatment of either acid soil based on the shoot P concentrations in the S or L lines (Fig. 5).

### Inheritance of acid soil rhizosheath size in wheat

**Fig. 6.**
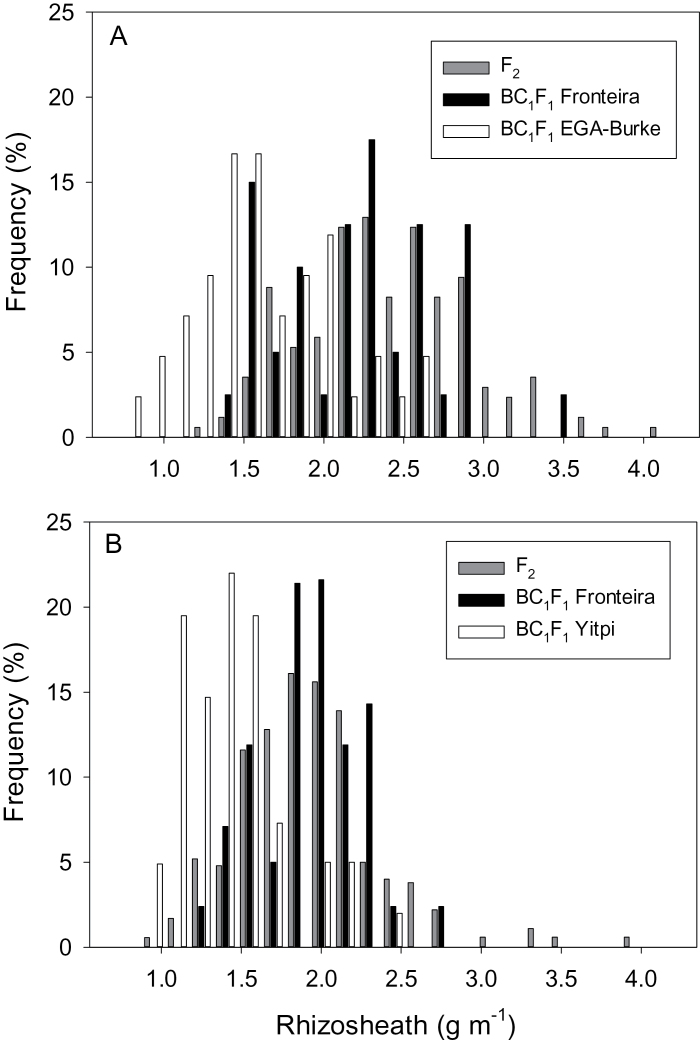
Frequency distributions for rhizosheath sizes measured on F_2_ and BC_1_F_1_ progeny in the Fronteira × EGA Burke and Fronteira × Yitpi populations.

Significant variation was observed between parents in generation means for rhizosheath size of seedlings grown on the acid soil (Table 2). Fronteira had significantly (*P*>0.01) larger rhizosheaths than either of the small rhizosheath parents EGA Burke and Yitpi. Differences in rhizosheath size among parents translated into significant (*P*<0.05) differences between progeny generations for rhizosheath size (Table 2). The F_1_ and F_2_ generation means were similar but both were smaller than the mid-parent mean for both crosses. Maternal genetic effects on rhizosheath size were small and not statistically significant (*P*>0.05) in each population (data not shown). Backcross-derived Fronteira progeny were on average larger (*P*<0.01) for rhizosheath than either backcross-derived EGA Burke or Yitpi progeny (Table 2). The distribution of F_2_ progeny values was Gaussian with parental values contained in the tails of each population (Fig. 6). The backcross progenies were also Gaussian in their distributions with evidence that the phenotype of the small rhizosheath parent was recovered in backcrosses using either EGA Burke or Yitpi. By contrast, when backcrossed to Fronteira the progeny did not recover the large rhizosheath of Fronteira.

**Table 2.** Parental, F_1_, F_2_ and BC_1_F_1_ means, and estimates of gene effects for root rhizosheath size for two wheat crosses

Generation	Fronteira/EGA Burke(g m^−1^)	Fronteira/Yitpi(g m^−1^)

Parent 1 (P_1_)	3.71 (0.11)	3.38 (0.10)
Parent 2 (P_2_)	1.68 (0.05)	1.25 (0.06)
F_1_	2.29 (0.09)	1.97 (0.08)
F_2_	2.38 (0.04)	1.93 (0.03)
BC_1_P_1_	2.22 (0.07)	1.94 (0.05)
BC_1_P_2_	1.63 (0.07)	1.45 (0.05)
l.s.d.	0.18	0.15
*m*	2.58**	2.20**
[*a*]	0.92**	0.94**
[*d*]	−0.58ns	−0.51ns
χ^2^ (*P*-value)^*a*^	1.98 (0.58ns)	3.88 (0.27ns)

Values in parentheses are the standard errors.

^*a*^ *P*-value for chi-square testing *H*_*O*_: adequacy of additive-dominance genetic model.

* and ** denote parameter estimates significantly different from zero at *P* = 0.05 and 0.01, respectively; ns denotes parameter estimates not significantly different at *P* = 0.05.

[*a*]: pooled additive genetic effect; [*d*]: pooled dominance genetic effect; l.s.d: least significant difference among generation means at *P* = 0.05; *m*: estimated mean.

**Fig. 7.**
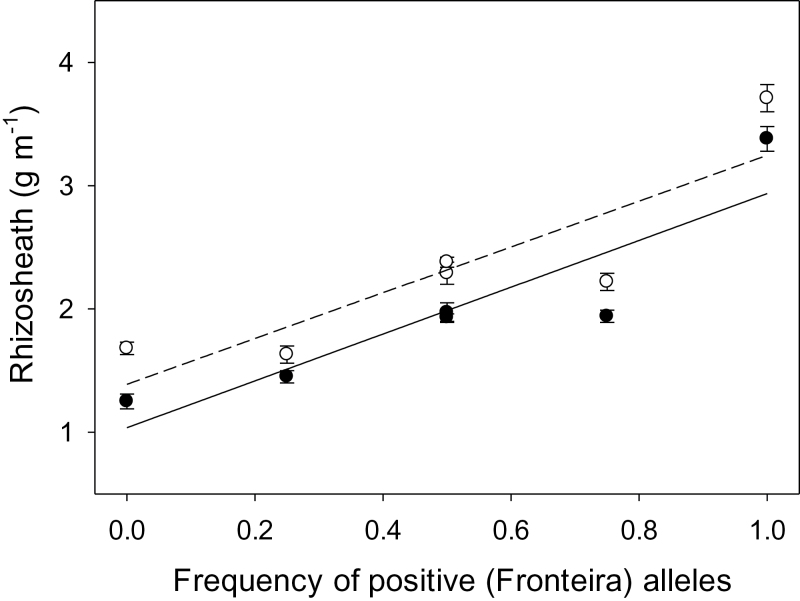
Relationship between generation means for frequency of the Fronteira (large rhizosheath donor) alleles measured for progeny of Fronteira × EGA Burke (open circles) and Fronteira × Yitpi (closed circles) crosses. Standard errors of each mean are also included. Least-squares relationships are: Fronteira × EGA Burke (dashed line), *Y*=1.38+1.86*X* (*r* ^2^=0.76, *P<*0.05); and Fronteira × Yitpi (solid line), *Y*=1.04+1.90*X* (*r* ^2^=0.81, *P<*0.01).

Increasing frequency of alleles from the rhizosheath donor was linearly associated with increases in rhizosheath sizes for generations in both crosses (Fig. 7). Coefficients of determination were high, ranging between 76 and 81%. The GMA for rhizosheath size for each cross revealed a largely additive-based genetic control for variation in rhizosheath size (Table 2). Goodness-of-fit tests revealed the additive model to be adequate for Fronteira by EGA Burke crosses (χ^2^=1.98; *P*>0.05) and Fronteira by Yitpi crosses (χ^2^=3.88; *P>*0.05) despite the deviations for F_1_ and F_2_ means from mid-parent, and rhizosheath means being smaller than expected for Fronteira-backcross progeny. In all cases, significant gene effects were repeatable across populations, and indicate that accumulation of positive alleles through selection is possible for rhizosheath size under additive genetic control (Fig. 7).

The among-generation variance for rhizosheath size was largest for the Fronteira/EGA Burke population (Table 3). However, proportionally larger residual variance for this population reduced repeatability to 0.70 consistent with repeatability in the Fronteira/Yitpi population of 0.74. Broad-sense heritabilities were estimated for rhizosheath size on a single-plant basis and were similar in both crosses (Table 3). Narrow-sense heritabilities were moderate in size reflecting their estimation on a single-plant basis and the proportionally larger additive gene effects observed for rhizosheath size. Reduced confidence in estimation of genotypic values contributed toward large differences in the estimates of numbers of factors contributing to genetic differences between the parents for rhizosheath size (Table 3). Despite these differences it appears that multiple genes contribute to the large rhizosheath of Fronteira.

**Table 3.** *Variance component (± standard errors), broad-sense* (H^*2*^ *) and narrow-sense* (h^*2*^ *) heritabilities (± standard errors), and estimated numbers of effective factors for rhizosheath size measured on two wheat populations*

Genetic parameter	Fronteira/EGA Burke	Fronteira/Yitpi
σ^2^_Genotype_	0.55±0.31*	0.46±0.27*
σ^2^_Residual_	0.24±0.02**	0.16±0.03*
*H* ^2^	0.59±0.09	0.58±0.08
*h* ^2^	0.33±0.08	0.47±0.06
No. effective factors	5.7	2.7

* and ** indicates parameter estimates are statistically different from zero at *P* = 0.05 and 0.01, respectively.

### QTL analysis of an EGA Burke × Fronteira RIL population

**Fig. 8.**
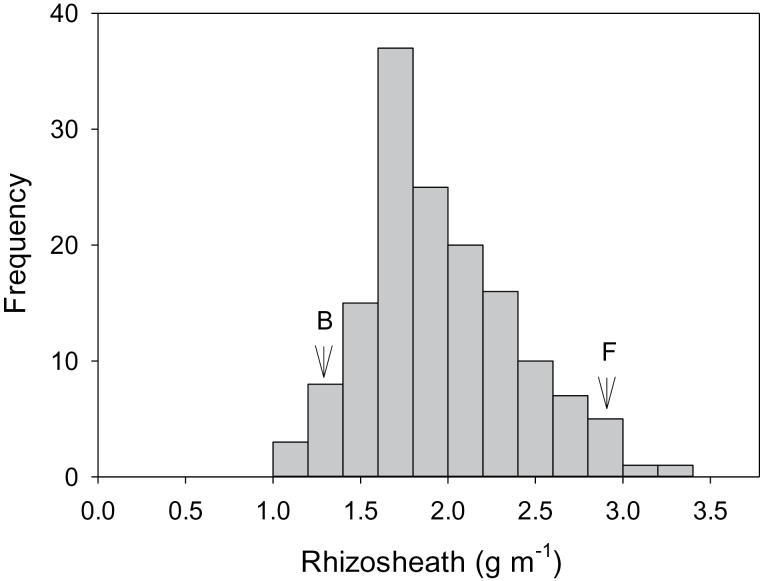
Frequency distribution of acid soil rhizosheath size for 139 RILS developed from an EGA Burke by Fronteira cross. Parental means for EGA Burke (B) and Fronteira (F) are indicated by arrows. Values for each line are the means of *n*=4–6 seedlings.

The RIL population derived from an EGA Burke × Fronteira cross encompassed the rhizosheath sizes of EGA Burke and Fronteira (Fig. 8). Heritability for acid soil rhizosheath size was 0.84 for the F_6_ RILs. Five QTL for acid soil rhizosheath size with LOD values greater than 3.0 were identified, which together accounted for 64% of the total genetic variance (Table 4). One major locus located on chromosome 1D accounted for over half of the genetic variance of rhizosheath size (34%). Other loci each contributing from 6.8 to 8.5% of the genetic variance for rhizosheath size were identified on chromosomes 3A, 3B, 6A and 7B (Table 4). All positive alleles for rhizosheath size were derived from the large rhizosheath donor parent Fronteira.

**Table 4.** *Chromosomal locations of QTL for acid soil rhizosheath size in EGA Burke × Fronteira F*_*6*_ *RILS*

Chromosome location	Molecular marker	Distance (cM)^*a*^	Allelic effect^*b*^ (g m^–1^)	Genetic variance (%)	LOD
1D	*D_contig14507_369*	179.25	0.209	34.1	15.78
3A	*Excalibur_c14216_692*	238.5	0.105	8.5	3.96
3B	*Ex_c70232_336*	266.9	0.095	7.0	3.27
6A2	*GENE-2724_97*	122.8	0.097	7.3	3.66
7B	*BobWhite_c8579_56*	144.6	0.094	6.8	3.06

^*a*^ Distance is the chromosomal distance from the tip of the chromosome.

^*b*^ Allelic effects are for Fronteira as the donor parent.

LOD: likelihood of odds.

## Discussion

The L and S lines used in this study were originally developed from phenotypic screens on the ferrosol without added nutrients (Delhaize *et al.*, 2012a). Here we show that the differences in rhizosheath sizes between lines were maintained on acid soils that had been fertilized and amended with both low and non-limiting P supplies (Fig. 1). The differences in rhizosheath sizes between the lines were attenuated compared with the previous work (Delhaize *et al.*, 2012a) and this can be attributed in part to the ameliorating effects of added nutrients on Al^3+^ toxicity. For the ferrosol, the high rate of P addition resulted in larger rhizosheaths compared with the low rate of P addition for all lines although differences between S and L lines were still observed (Fig. 1B). This is consistent with P detoxifying a proportion of the Al^3+^ although this effect was not observed for the kandasol. When soil pH was increased by liming, rhizosheath sizes of all lines were increased markedly and although some lines differed for rhizosheath size, they did not separate into L and S groups (see Supplementary Fig. S1 at *JXB* online). This is consistent with previous findings that the differences in rhizosheath size of the lines were due to differences in the Al^3+^ tolerance of root hairs (Delhaize *et al.*, 2012a). Increasing the soil pH detoxifies the Al^3+^ and results in similar root hair lengths for all lines.

Previous studies have concluded that root hairs are an important factor for PAE of plants, but these studies have either compared wild type plants with mutants that lack root hairs entirely (Bates and Lynch, 2000, 2001; Gahoonia *et al.*, 2001; Gahoonia and Nielsen, 2003; Chen *et al.*, 2005; Zheng *et al.*, 2011; Brown *et al.*, 2012; Haling *et al.*, 2013) or compared genetically unrelated genotypes within species (Gahoonia *et al.*, 1997; Krasilnikoff *et al.*, 2003; Gahoonia and Nielsen, 2004; Zhu *et al.*, 2010; Vandamme *et al.*, 2013). Comparing mutants that lack root hairs with wild type plants has been useful in defining the role of root hairs in processes such as P uptake, but is not representative of the natural variation for root hair length within a species. Even when genotypes within a species with varying root hair length are assessed for PAE, the genotypes typically have not been backcrossed and likely differ in a range of other root attributes so that the contribution of root hairs towards PAE is uncertain. Here we show that improved PAE is associated with large rhizosheaths (long root hairs) in wheat NILs grown on acid soils. The improved PAE can be attributed to the root hairs since multiple NILs were developed by backcrossing and when they were grown on limed soils where differences in rhizosheath sizes were abolished, they did not differ in their PAE. The one exception was cv Fronteira, which had the greatest shoot biomass of all lines at both low and high P regardless of whether the soil was limed or not (Fig. 2). This indicates that Fronteira has traits in addition to long root hairs that contributed to its greater PAE and illustrates the value of using NILs for attributing PAE to a particular trait. An additional trait contributing to the PAE of Fronteira might have been the inherent vigour from Fronteira possessing the wild type *Rht* alleles that were previously shown to confer improved vigour and greater PAE (Botwright *et al.*, 2005; Ryan *et al.*, 2015). All of the other lines used in our study have the *Rht-B1b* allele conferring a semi-dwarf habit derived from EGA Burke.

Interestingly, the large rhizosheath trait conferred greater shoot biomass at both low and high P treatments. The high P treatments were chosen to be non-limiting for growth so it was unlikely that the greater biomass was due to improved PAE. The P concentrations in the high P treatments of the S lines were unlikely to have been limiting for growth with about 0.45% in shoots of plants grown on the kandasol and almost 0.80% in shoots of plants grown on the ferrosol (Fig. 5). An alternative explanation for the increased biomass of L lines at high P is that the large rhizosheaths provided other benefits. One possibility is that the larger rhizosheath improved water uptake. The water regime was not intended to restrict growth, but soil water content would have varied during the experiments particularly towards the end of the growth period when plants were at their largest. Water was applied every two days, but high rates of transpiration would have temporarily depleted soil water. The role of rhizosheaths in maintaining moisture around roots growing in soil (Young, 1995) and the proposed role of root hairs in effective uptake of soil moisture (Segal *et al.*, 2008) might have contributed towards the greater biomass of the large rhizosheath lines grown with high P supplies.

Analysis of the genetic control of the acid rhizosheath trait by GMA firstly identified the trait to be highly heritable, and secondly, that multiple loci were contributing to expression of the trait. This was subsequently confirmed in a QTL analysis of F_6_ RILs with five loci identified with LODs of greater than 3.0. One major locus on chromosome 1D on its own accounted for about half of the genetic variance. A previous study identified six loci for rhizosheath size of wheat seedlings grown on non-acid soils but none of these QTL co-located to the acid rhizosheath QTL identified here (Delhaize *et al.*, 2015). This is consistent with the genes protecting root hairs from Al^3+^ toxicity not confering long root hairs on non-acid soils. That different sets of genes contribute to each of Al^3+^ tolerance and long root hairs on non-acid soils was shown by the markedly small rhizosheaths of RILs derived from a multi-parent population when the same lines varied considerably for rhizosheath size on a non-acid soil (Delhaize *et al.*, 2015). None of the acid rhizosheath QTL were located on chromosomes 4D and 4B where well-characterized genes for Al^3+^ tolerance of root growth are located (Delhaize *et al.*, 2012b). One acid rhizosheath locus was located on chromosome 3B where an Al^3+^ tolerance locus for root growth has been described (Navakode *et al.*, 2009), but it remains to be established that these are the same genes.

This study has shown that it is possible to develop wheat lines with improved PAE based on a phenotypic screen for rhizosheath size as a surrogate for root hair length. However, despite transferring a large proportion of the trait, none of the backcrossed lines had rhizosheaths as large as Fronteira, the donor parent. The realized heritability estimated for the parent and offspring rhizosheath size assessments was *h*_R_^2^=0.39. Together with the single-plant heritabilities reported for the GMA, the consistently lower narrow-sense heritabilities for rhizosheath size indicates the potential for genetic gain based on phenotypic screening, but only with sufficient replication to improve precision on progeny means. The effectiveness of increased replication on heritability is illustrated by the QTL analysis that produced a relatively high heritability of 0.84 through the use of six replicates. A crossing programme that relied on phenotypic screens would require that many lineages of selections be maintained to ensure that lines with the largest acid rhizosheath size are developed. The availability of molecular markers linked to the QTL should now facilitate the crossing to ensure efficient and effective transfer of this trait to acceptor lines with far fewer genetic crosses.

In conclusion, we show that root hair length is one factor that can improve the PAE of wheat grown on acid soils in pot trials. Future work will need to establish whether the longer root hairs provide a benefit to grain yields in field trials and it is likely that other root attributes will be required in combination with long root hairs for further improvements in PAE. Clearly the major Al^3+^ tolerance gene for root growth (*TaALMT1*) is critical for ensuring root growth of wheat on acid soils with a direct benefit to PAE by allowing effective soil exploration (Delhaize *et al.*, 2009). All the germplasm used in the current study are ‘fixed’ for *TaALMT1* and their roots are considered to be tolerant of acid soils. Our work has shown that root hairs provide a PAE benefit to wheat grown on acid soil in addition to any benefit conferred by *TaALMT1*.

## Supplementary data

Supplementary data are available at *JXB* online.

Figure S1. Rhizosheath size of EGA Burke, Fronteira and selected BC_3_ NILS grown on non-acid soils.

Table S1. Shoot P concentration (% of dry weight) and shoot P content (mg plant^–1^) of EGA Burke, Fronteira and BC_3_ NILS with large rhizosheath (LR) or small rhizosheath (SR) after 28 d growth on a limed ferrosol with 250 (250 P) or 2000mg P kg^–1^ (2000 P) added.

Supplementary Data
