# Supplementary material for: Rhizosheaths on wheat grown in acid soils: phosphorus acquisition efficiency and genetic control
Source: J Exp Bot. 2016 Feb 11;67(12):3709–18. doi: 10.1093/jxb/erw035 (PMC4896358; doi:10.1093/jxb/erw035)
Supplement: Supplementary Data [file supp_67_12_3709__index.html]

Rhizosheaths on wheat grown in acid soils: phosphorus acquisition efficiency and genetic control — Rhizosheaths on wheat grown in acid soils: phosphorus acquisition efficiency and genetic control — Supplementary Data 

# Rhizosheaths on wheat grown in acid soils: phosphorus acquisition efficiency and genetic control

## Supplementary Data

Data files

- Supplementary\_Figure\_S1\_Table\_S1.pdf - Supplementary Data
