# Supplementary material for: Rhizosheaths on wheat grown in acid soils: phosphorus acquisition efficiency and genetic control
Source: J Exp Bot. 2016 Feb 11;67(12):3709–18. doi: 10.1093/jxb/erw035 (PMC4896358; doi:10.1093/jxb/erw035)
Supplement: Supplementary Data [file supp_erw035_Supplementary_Figure_S1_Table_S1.pdf]

Rhizosheaths on wheat grown in acid soils: phosphorus acquisition efficiency and genetic control

Richard A. James, Chandrakumara Weligama, Klara Verbyla, Peter R. Ryan, Gregory J. Rebetzke, Allan Rattey, Alan E. Richardson, Emmanuel Delhaize

Supplementary Figure S1 and Table S1

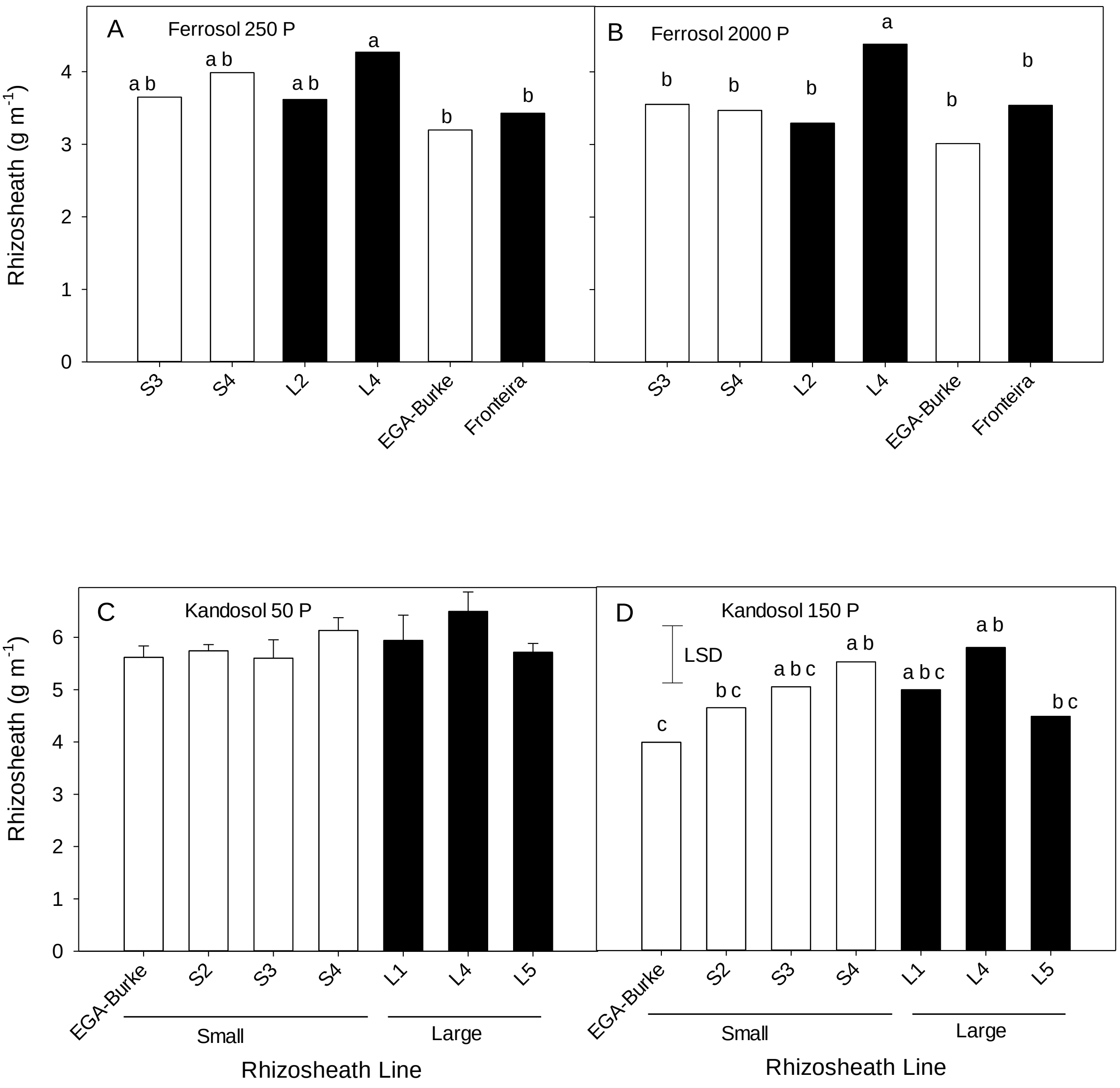

Figure S1. Rhizosheath size of EGA Burke, Fronteira and selected BC_3 NILS grown on non-acid soils. The lines were originally selected for small (S lines, empty bars) or large (L lines, filled bars) rhizosheaths on acid soils. The acid ferrosol (A, B) and the acid kandosol (C, D) with a responsive (A,C) or a non-responsive (B,D) P rate added were limed to pH 5.5. Values are means (n=6) and different letters indicate significant differences (P = 0.05) between genotypes.

Table S1. Shoot P concentration (% of dry weight) and shoot P content (mg plant^{-1}) of EGA Burke, Fronteira and BC_3 NILS with large rhizosheath (LR) or small rhizosheath (SR) after 28 d growth on a limed ferrosol with 250 (250 P) or 2000 mg P kg^{-1} (2000P) added. Values are means (n=4) and different letters indicate significant differences between genotype means as determined from a two-way ANOVA. Shown is the least significant difference (LSD) at P=0.05 and n.s. denotes no significant differences.

Genotype		Shoot P concentration (%)		Shoot P content (mg plant^{-1})	
		250 P	2000 P	250 P	2000 P
EGA Burke		0.407	0.650a	3.73	8.08a
Fronteira		0.412	0.957c	4.41	12.48c
LR NILS	L2	0.396	0.865b	3.61	9.80b
	L4	0.382	0.958c	3.23	11.24c
SR NILS	S3	0.415	0.837b	3.64	9.82b
	S4	0.431	0.741ab	4.51	8.38a
LSD		n.s.	0.074	n.s.	1.39
